# Optical tomography dynamics induced by qubit-resonator interaction under intrinsic decoherence

**DOI:** 10.1038/s41598-022-21348-4

**Published:** 2022-10-13

**Authors:** A. -B. A. Mohamed, H. Eleuch

**Affiliations:** 1 Department of Mathematics, College of Science and Humanities in Al-Aflaj, Prince Sattam bin Abdulaziz University, Al-Aflaj, Saudi Arabia; 2 Department of Mathematics, Faculty of Science, Assiut University, Assiut, Egypt; 3 Department of Applied Physics and Astronomy, University of Sharjah, Sharjah, 27272 United Arab Emirates; 4 Department of Applied Sciences and Mathematics, College of Arts and Sciences, Abu Dhabi University, Abu Dhabi, 59911 United Arab Emirates; 5 Institute for Quantum Science and Engineering, Texas A &M University, College Station, TX 77843 USA

**Keywords:** Quantum information, Qubits, Theoretical physics

## Abstract

A superconducting circuit with a qubit and a resonator coupled via a two-photon interaction is considered. When the resonator is initially in a superposition of coherent states, optical tomography and quantum coherence dynamics are examined in the context of intrinsic decoherence. The results reveal that optical tomography is a good quantifier of the quantum coherence produced by the qubit-resonator interaction. The effects of qubit-resonator detuning and intrinsic decoherence on the dynamics of optical tomography distributions for coherent and even coherent states are investigated. The dynamics of optical tomography distributions are highly dependent on detuning and intrinsic decoherence. Our numerical simulations reveal that there is a relation between the optical tomography and the generated quantum coherence. When the qubit-resonator detuning and intrinsic decoherence are augmented, the amplitude and intensity, as well as the structure of the optical tomography, change substantially.

## Introduction

A tomographic quantifier is a ubiquitous tool for estimating the physical properties of quantum states, and its procedure depends on the density matrix of the quantum state^1^. The optical homodyne tomography is a monotonic relation between the density operator and the probability distributions of the coherent field states^2^, which enables all conceivable information about the quantum states to be extracted^3–5^. Therefore, many nonclassical light states can be characterized using the optical homodyne tomography. Optical tomography is a critical method for testing any quantum information processing implementation^6,7^. It has been investigated theoretically and experimentally for neural-network quantum states^8^, maximally entangled states of beam splitter modes^9^, *q*-deformed coherent states^10^, and a single electron spin state associated with a nitrogen-vacancy center^11^. The optical homodyne tomography is used to analyze revivals in a Kerr medium^12^.

More attention has been paid to realize artificial light-matter interactions^13^ such as: superconducting circuits^14,15^ quantum dot^16^ trapped ions^17^, to generate useful nonclassical effects such as coherence, squeezing, and quantum correlations. Superconducting systems, in particular, enable a significant flexibility in the design of superconducting artificial qubits interacting with resonators^18^. Such systems have opened the door for a number of theoretical studies on theses artificial qubits and the nonlinear interactions^19^. Two-photon transition has been predicted by Goeppert-Mayer^20^. This effect has been widely utilized in fluorescence microscopy^21^, and dichromatic laser pulses^22^. Two-photon quantum Rabi model was implement based on proposed superconducting circuit in a solid-state device^15^.

The potential of establishing quantum computing and information in the context of superconducting circuits has been investigated using Wigner tomography of coherent state superpositions and a fixed-frequency superconducting transmon qubit coupled to a waveguide cavity resonator^23–25^. Recently, superconducting transmon qubits and two photonic qubits were utilized in experiments using quantum tomography to: realize an architecture of two coupled logical qubits^26^, and to demonstrate controlled-phase gates between two Error-correctable photonic qubits^27^, as well as to reveal quantum correlations in bi-mode cavity^28^.

Optical homodyne tomography distributions of a quantum state are highly vulnerable to decoherence and dissipation resources^12^ due to the strong relation between the nonclassically loss and the decoherence effect. In this paper, we investigate the dynamics of the proposed qubit-resonator interactions using the Milburn motion equation, which governs the dynamics of the system in the presence of intrinsic decoherence^29^. The intrinsic decoherence model has been used to investigate the dynamics of quantum information resources in numerous systems^30–33^. The intrinsic decoherence was theoretically investigated^29^ and implemented in a linear array of coupled trapped-ion qubits^34^ without any explicit coupling to the surrounding environment. This form of decoherence comes into play in closed qubit-resonator interactions. The intrinsic decoherence results in a quantum coherence loss without loss of energy. The dynamics of the system is described in this case by the Milburn equation. Others approaches are governed by Master equations, describing system-reservoir interactions (open quantum systems)^35,36^, which lead naturally to decoherence as the information is immediately lost after evolution. The intrinsic decoherence differs from the dissipation which occurs in open quantum systems^37–39^ that interacts with the surrounding environment.

In various superconducting circuits^40^ and Nitrogen-Vacancy Centers^41^, the intrinsic decoherence processes have been realistically controlled. The intrinsic decoherence time in isolated quantum systems was investigated, and it was discovered that it is dependent on both the system size and the disorder strength^34^. Quantum error detection reduces the influence of intrinsic decoherence in a superconducting circuit^42^. The phase coherence in microcavity polariton condensate^43^ and Josephson qubits^44^ is controlled by intrinsic decoherence processes.

Previously, the dynamics of optical homodyne tomography were restricted to specific models^9,12^, that ignoring the presence of decoherence and dissipation effects. Optical tomography and coherence dynamics in the context of a superconducting circuit are investigated in this paper. The resonant two-photon interaction of a qubit coupled to a resonator, whose state is initialized in a coherent state or a superposition of coherent states, models the relevant dynamics. In addition, the influence of qubit-resonator detuning and intrinsic decoherence on the dynamics of the optical tomography distributions for the selected system’s states is the focus of this study. As compared to the optical tomography considered previously, here the optical tomography distribution is explored with the qubit-resonator interaction in the presence of the intrinsic decoherence.

The paper is organized as follows: In the “Qubit-resonator system with intrinsic decoherence model” section, the qubit-resonator system with intrinsic decoherence model is presented with its analytical solutions. In “Optical tomography distribution, Optical tomography dynamics” sections, the dynamics of the optical tomography distribution for the generated time-dependent resonator state is illustrated. “Conclusions” section is dedicated to the conclusion.

## Qubit-resonator system with intrinsic decoherence model

We consider a two-photon qubit-resonator model that describes a qubit and a resonator coupled via two-photon transition. The model can be realized as a superconducting circuit consisting of a dc-SQUID with two identical junctions inductively coupled to a superconducting flux qubit. The Hamiltonian of the two-photon qubit-resonator with the quadratic coupling is given by ($\hbar =1$)^45^

1 $$\begin{aligned} {\hat{H}}&= \omega _{SQ} \left( {\hat{\psi }}^{\dagger } {\hat{\psi }}+\frac{1}{2}\right) +\frac{1}{2}\omega _{q}{\hat{\sigma }}_{z} -\frac{\pi }{4} \tan \left( \frac{\pi \Phi _{DC}}{\Phi _{0}}\right) \frac{M I_{p}}{\Phi _{0}}\omega _{SQ} ({\hat{\psi }}+ {\hat{\psi }}^{\dagger })^{2} {\hat{\sigma }}_{x}. \end{aligned}$$

$\Phi _{DC}$ represents an externally applied static flux. $\omega _{q}$, *M*, and $I_{p}$ represent respectively the frequency, the mutual inductance, and the persistent current states of the qubit-SQUID. $\omega _{SQ}$ is SQUID mode frequency with the annihilation operator ${\hat{\psi }}$. $|0\rangle$ and $|1\rangle$ are the qubit ground and excited states. ${\hat{\sigma }}_{x}$ and ${{\hat{\sigma }}}_{z}$ design the Pauli qubit density matrices. Here, we focus on the SC regime, where the coupling strength is small in comparison to the qubit and cavity eigenfrequencies, but greater than all dissipation rates. In the SC regime, the counter-rotating terms ${\hat{\sigma }}_{-} {\hat{\psi }}^{2} + H.c$. and ${\hat{\sigma }}_{x}{\hat{\psi }}^{\dagger } {\hat{\psi }}$, rotating at frequencies $2\omega _{q} + \omega _{SQ}$ and $\omega _{q}$, respectively, can then be neglected. The Hamiltonian can be written as^45^

2 $$\begin{aligned} \!\!\!\!\!{\hat{H}}&= \omega _{SQ} ({\hat{\psi }}^{\dagger } {\hat{\psi }}+{\hat{\sigma }}_{z}) + \!\!\frac{\delta }{2} {\hat{\sigma }}_{z}+ \!\!\lambda ({\hat{\psi }}^{2}|1\rangle \langle 0|+ |0\rangle \langle 1|{\hat{\psi }}^{2\dagger }). \end{aligned}$$

$\lambda =-\frac{\pi }{4} \tan (\frac{\pi \Phi _{DC}}{\Phi _{0}}) \frac{M I_{p}}{\Phi _{0}}\omega _{SQ}$. $\delta =\omega _{q}-2\omega _{SQ}$ represents the qubit-resonator detuning, for the off-resonance case $\delta \ne 0$. In the spanned space of qubit-resonator states $\{|\varpi _{1}\rangle =|1, n \rangle , |\varpi _{2}\rangle =|0, n+2\rangle \}$, the dressed-states of the Hamiltonian of Eq. (2) are given by

3 $$\begin{aligned} |S_{1}^{n}\rangle&= \sin \zeta _{n}|\varpi _{1}\rangle + \cos \zeta _{n}|\varpi _{2}\rangle , \nonumber \\ |S_{2}^{n}\rangle&= \cos \zeta _{n}|\varpi _{1}\rangle -\sin \zeta _{n}|\varpi _{2}\rangle , \end{aligned}$$

where

4 $$\begin{aligned} \zeta _{n}&= \sin ^{-1} \sqrt{(X_{n}+\delta )/2X_{n}}, \nonumber \\ X_{n}&= \sqrt{(\omega _{q}-2\omega _{SQ})^{2}+\lambda ^{2}(n+1)(n+2)}. \end{aligned}$$

The energy eigenvalues have the following expressions

5 $$\begin{aligned} V^{n}_{1}&= \omega _{SQ}(n+2)+X_{n}, \nonumber \\ V^{n}_{2}&= \omega _{SQ}(n+2)-X_{n}. \end{aligned}$$

For the ground state energy $|0,0\rangle$, the energy eigenvalue is $V_{0}=-\frac{\delta }{2}$.

Here, the time evolution of the qubit-resonator interactions and the decoherence intrinsic effect on the dynamics of the qubit-resonator is explored by the Milburn’s equation^29^,

6 $$\begin{aligned} \frac{d {\hat{\rho }}(t)}{d t}&= -i[{\hat{H}},{\hat{\rho }}(t)]-\frac{\gamma }{2}[{\hat{H}},[{\hat{H}},{\hat{\rho }}(t)]], \end{aligned}$$

where $\gamma$ denotes intrinsic decoherence, in which quantum coherence is lost naturally as the system evolves. This intrinsic decoherence process has been introduced as propose a modification of the standard quantum mechanics evolution, built on the hypothesis that the qubit-resonator system dynamics under a stochastic succession of identical unitary transformations for sufficiently short time steps rather than a continuous unitary evolution^29^. In the intrinsic-decoherence models, the off-diagonal elements of the density matrix are intrinsically suppressed in the energy eigenstate basis, consequently, intrinsic decoherence is realized without the dissipation.

To find a particular solution for the differential Milburn’s equation, we assumed that the initial qubit state is in the excited state $|1\rangle \langle 1|$. While the initial resonator state is a superposition of the two “opposite” coherent states $|\pm \alpha \rangle$ (these two superposed states are $\pi$ phased), the initial resonator reduced density matrix is given by

7 $$\begin{aligned} \!\!\!\!\!\!{\hat{\rho }}_{R}(0)&= \frac{1}{A}(|\alpha \rangle +r|-\alpha \rangle ) (|\alpha \rangle +r|-\alpha \rangle )^{\dagger } \end{aligned}$$

8 $$\begin{aligned}&= \sum _{m,n=0}P_{m}P^{*}_{n}|m\rangle \langle n|, \end{aligned}$$

*A* designs the normalization factor, and the photon distribution function is given by

9 $$\begin{aligned} P_{n}=\frac{[1+r(-1)^{n}]\alpha ^{n}e^{-\frac{1}{2}|\alpha |^{2}}}{\sqrt{(1+r^{2}+2r\langle \alpha |-\alpha \rangle )n!}}. \end{aligned}$$

The values $r= 0, 1, -1$ correspond to a coherent state, an even coherent state, and an odd coherent state, respectively.

To solve the Eq. (6), the initial qubit-resonator state is reexpressed by using the dressed-states and the energy eigenvalues of the Hamiltonian of Eq. (2) that satisfies the condition of the eigenvalue-problem: ${\hat{H}}|S_{k}^{n}\rangle =V^{n}_{k}|S_{k}^{n}\rangle (k=1,2)$, $V^{n}_{k}$ correspond to the eigenvalues. Therefore, using the Eq. (6), the density matrix dynamics of the dressed states, $S^{mn}_{kl}(t)=|S_{k}^{m}\rangle \langle S_{k}^{n}|$, which have the following expression

10 $$\begin{aligned} S^{mn}_{kl}(t)&= D^{mn}(t) e^{-i\lambda (V^{m}_{i}-V^{n}_{j})t}|S_{k}^{m}\rangle \langle S_{l}^{n}|, \end{aligned}$$

where $D^{mn}(t)=e^{-\frac{\gamma }{2}(V^{m}_{k}-V^{n}_{l})^{2}t}$ represents the intrinsic decoherence term. Then, using the initial state and the Hamiltonian (2), the particular solution of Eq. (6) is then

11 $$\begin{aligned} {\hat{\rho }}(t)&= \sum _{m,n=0}P_{m}P^{*}_{n}[\sin \zeta _{m} \sin \zeta _{n} H^{mn}_{11}S^{mn}_{11}(t) +\sin \zeta _{m} \cos \zeta _{n} H^{mn}_{12} S^{mn}_{12}(t)\nonumber \\&+\cos \zeta _{m} \sin \zeta _{n} H^{mn}_{21} S^{mn}_{21}(t)+\cos \zeta _{m} \cos \zeta _{n} H^{mn}_{22}S^{mn}_{22}(t)], \end{aligned}$$

where $l=C$ for the initial coherent state and $l=Ec$ for the initial even coherent state with the abbreviations:

12 $$\begin{aligned} H^{mn}_{kl}= e^{-i(V^{m}_{k}-V^{n}_{l}) t-\frac{\gamma }{2}(V^{m}_{k}-V^{n}_{l})^{2}t}. \end{aligned}$$

The study of the dynamics of the optical tomography is mainly built on the resonator reduced density matrix,

13 $$\begin{aligned} \!\!\!\!\!\!\!{\hat{\rho }}_{R}(t)=\!\!\!\sum _{m,n=0}P_{m}P^{*}_{n}(\chi _{1}|m\rangle \langle n| +\chi _{2}|m+2\rangle \langle n+2|), \end{aligned}$$

with

$$\begin{aligned} \chi _{1}&= \sin ^{2}\zeta _{m}\sin ^{2}\zeta _{n} H^{mn}_{11}+\sin ^{2}\zeta _{m} \cos ^{2}\zeta _{n}H^{mn}_{12}\\&\quad+\cos ^{2}\zeta _{m} \sin ^{2}\zeta _{n}H^{mn}_{2l}+\cos ^{2}\zeta _{m}\cos ^{2}\zeta _{n}H^{mn}_{22}, \\ \chi _{2}&= \sin \zeta _{m} \sin \zeta _{n}\cos \zeta _{m}\cos \zeta _{n}(H^{mn}_{11}- H^{mn}_{12} -H^{mn}_{21}+H^{mn}_{22}). \end{aligned}$$

The optical tomography dynamic of the produced field states will be explored in the next section based on the reduced cavity density matrix ${\hat{\rho }}^{f}(t)$.

**Figure 1:**
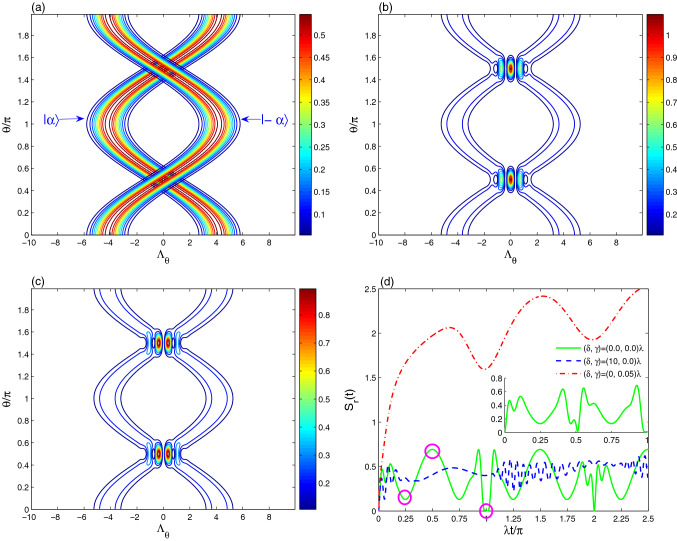
Optical tomography contour of the coherent state is illustrated in (**a**), the even coherent state in (**b**), and the odd coherent state in (**c**) for $|\alpha |^{2}=9$. In (**d**), the dynamics of the resonator field entropy under the decoherence and detuning effects is plotted for the coherent state, and the even coherent state in the sub-figure.

## Optical tomography distribution

The optical tomography for the time-dependent resonator state is calculated by the resonator reduced density matrix ${\hat{\rho }}_{R}(t)$ as^46^:

14 $$\begin{aligned} T(\Lambda _{\theta },\theta )=\langle {\hat{\Lambda }}_{\theta }|{\hat{\rho }}|{\hat{\Lambda }}_{\theta }\rangle , \end{aligned}$$

where $|{\hat{\Lambda }}_{\theta }\rangle$ designs the eigenstate of the homodyne quadrature operator

15 $$\begin{aligned} {\hat{\Lambda }}_{\theta }=\frac{1}{\sqrt{2}}({\hat{\psi }}\exp -i\theta +{\hat{\psi }}^{\dagger }\exp i\theta ), \end{aligned}$$

with eigenvalue $\Lambda _{\theta }$ and the local oscillator phase $\theta$. ${\hat{\psi }}$ and ${\hat{\psi }}^{\dagger }$ are the single-mode field operators. Equation (14) verifies the normalization relation

16 $$\begin{aligned} \int T(\Lambda _{\theta },\theta )d\Lambda _{\theta }=1. \end{aligned}$$

After writing the single-mode cavity field density matrix ${\hat{\rho }}_{R}(t)$ of Eq. (13) in number state representation as: ${\hat{\rho }}_{R}(t)=\sum \limits _{m,n =0}^{\infty }\rho ^{mn}_{R}|m\rangle \langle n|$, we use this representation into (14). Therefore, the expression of the optical tomography is then

17 $$\begin{aligned} T(\Lambda _{\theta },\theta ,t)&= \sum \limits _{m,n =0}^{\infty }\rho ^{mn}_{R}(t) Z_{m}(\Lambda _{\theta },\theta ) Z_{n}^{*}(\Lambda _{\theta },\theta ), \end{aligned}$$

with

$$\begin{aligned} Z_{m}(\Lambda _{\theta },\theta )=\langle \Lambda _{\theta },\theta |m\rangle&= \frac{e^{-\frac{X_{\theta }^{2}}{2}} }{\pi ^{\frac{1}{4}}}\frac{e^{-im\theta }H_{m}(\Lambda _{\theta })}{\sqrt{m!2^{m}}}. \end{aligned}$$

where $H_{n}(.)$ indicates the order *n* Hermite polynomial. Equation (17) describes the time dynamics of optical tomography in a system including the qubit-resonator interaction described by the Eq. (2). In the sections that follow, we will examine the temporal evolution of optical tomography for various types of initial coherent states in the presence of intrinsic decoherence.

**Figure 2:**
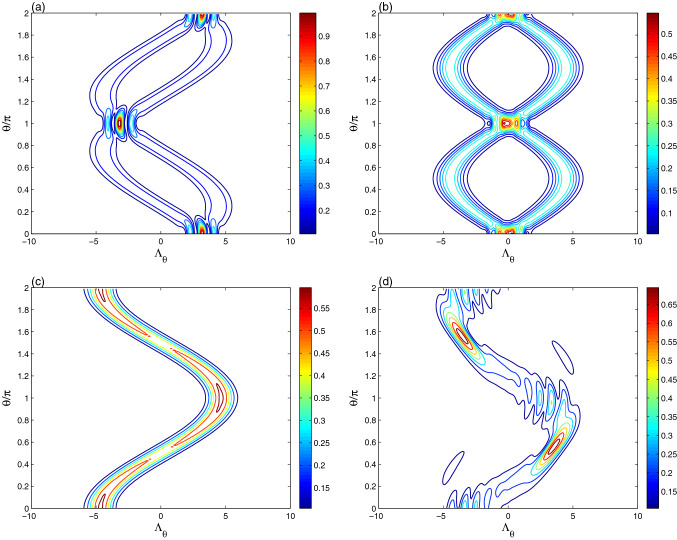
The dynamics of the optical tomography contour of the initial coherent state for $|\alpha |^{2}=9$ with different times is represented at $\lambda t= \frac{1}{4}\pi$ in (**a**), $\lambda t= \frac{1}{2}\pi$ in (**b**), and $\lambda t=\pi$ in (**c**). In (**d**), the optical tomography of (**c**) under the detuning effect $\delta =10\lambda$ is plotted.

## Optical tomography dynamics

In Fig. 1, the optical tomography contour $T(\Lambda _{\theta }, \theta , t=0)$ of the coherent states (CSs), the even coherent state (ECS) and the odd coherent (OCS) are illustrated. Figure 1a shows that the optical tomography of the two “opposite” coherent states $|\pm \alpha \rangle$. The optical CS-tomography of $|\alpha \rangle$ behaves similar to sine-curve function with $2\pi$-periodic and constant amplitudes in the $\theta -\Lambda _{\theta }$-plane. In the case, for the increase of the local oscillator phase angle ($\theta \rightarrow 2\pi$), the optical CS-tomography starts from the point $(\Lambda _{\theta }, \theta )= (|\alpha |\sqrt{2}, 0)\simeq (4.2426,0)$ increasing towards the point $(-4.2426, \pi )$, and then it decreases towards the point $(4.2426, 2\pi )$. While the optical CS-tomography of $|-\alpha \rangle$ behaves as cosine-curve function. The optical tomographies of $|\pm \alpha \rangle$ have two opposite distributions which are depending on the coherent intensity $|\alpha |$.

Figure 1b illustrates that the optical ECS-tomography contour is appeared as intersected regular cosine-curve paths. The ECS-tomography is also depicted for the superposition of the two “opposite” coherent states $|\pm \alpha \rangle$. The Insert-Colobar shows that the tomography distribution has constant amplitudes in the $\Lambda _{\theta }-\theta$-plane except in the interference regions. The distribution of the amplitudes is different and they are enhanced as the ECS-tomography tends to its center points $(\theta , \Lambda _{\theta })=(\frac{1}{2}(2n+1),0) (n=0, 1)$. The results of the Fig. 1b,c show that the optical tomographies of the ECS and OCS differ only at interference regions. This difference depends on the initial coherent field intensity. It was proven that, for large field intensities, the non-classical properties of the even and odd coherent states are approximatively the same^9,47^. Therefore, our investigations are focused on the coherent states and the even coherent state.

To investigate the relation between the optical tomography and the resonator quantum coherence dynamics, the time evolution of the resonator field entropy under the decoherence and detuning effects are shown in Fig. 1d. The resonator entropy is calculated by:

18 $$\begin{aligned} S_{r}(t)=-\sum _{n}\pi ^{i}_{n}\,\,\ln \pi ^{i}_{n}, \end{aligned}$$

where $\pi ^{i}_{n}$ represent the eigenvalues of the resonator reduced-density matrix ${\hat{\rho }}_{R}(t)$ of Eq. (13). Solid curves of Fig. 1d indicates that the qubit-resonator interactions annihilate the coherence of the resonator field (i.e., the generated mixedness of the resonator increases). The sub-figure of Fig. 1d illustrates the oscillatory behaviour of the even coherent state during the first $\pi$-period. Note that at $\lambda t= \frac{1}{2}\pi$, the resonator can be in a mixed state if its initial state was in the coherent state, and it can be in a pure state if it is initially in the even coherent state. The regularity of the generated resonator mixedness is disappeared after considering the qubit-resonator detuning effect. The mixedness is enhanced with more oscillations. Dot and dash-dot curves of Fig. 1d show that the resonator entropy grows due to the intrinsic decoherence, in other words, the generated resonator mixedness is enhanced.

Figure 2 illustrates the dynamics of the optical tomography of the coherent state $|\alpha \rangle$ under the qubit-resonator interactions in the resonant case $\delta =0$. At $t =\frac{1}{4}\pi$ (the resonator state has partial coherence), the optical CS-tomography evolves to two symmetrical distribution branches around the axis $\theta =\pi$, see Fig. 2a. The maximum values of the interference regions are larger than those of the initial distribution of Fig. 1a. For $t =\frac{1}{2}\pi$, the generated optical CS-tomography has two irregular sinusoidal paths, which are similar to the shape of the initial distribution of the even coherent states. The optical tomography evolution can be used as a good indicator of the field resonator mixedness generated by the qubit-resonator interactions.

This result is confirmed for the optical CS-tomography displayed at $\lambda t =\pi$ (the resonator state is in a pure state, see Fig. 1d). The generated resonator state presents optical tomography with cosine-curve function which is similar to of the coherent state $|-\alpha \rangle$, see Fig. 1a. This means that the generated pure resonator state is the coherent state $|-\alpha \rangle$.

Figure 2d shows the dependence of the optical tomography of the coherent state $|\alpha \rangle$, generated at $\lambda t =\pi$, with the detuning $\delta =10\lambda$. The amplitudes and the intensity of the optical tomography are enhanced due to the increase of the qubit-resonator detuning.

**Figure 3:**
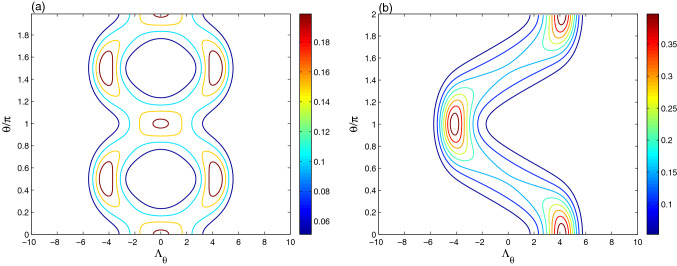
The effect of the intrinsic decoherence is illustrated on the optical tomography dynamics of the Fig. 2b,c for $\gamma =0.05\lambda$.

Figure 3, displays the intrinsic decoherence effect $\gamma =0.05\lambda$ on the generated optical CS-tomography at the different times $\lambda t= \frac{1}{2}\pi$ and $\lambda t=\pi$ of Fig. 2b,c. The intrinsic decoherence effect leads to: (1) the amplitudes and the contour intensity of the CS-optical tomography are reduced at different times. At $\lambda t= \frac{1}{2}\pi$ and $\lambda t=\pi$, the sizes of the distribution shape change due to the intrinsic decoherence effect.

In the case of resonance, we observe that optical tomography for a periodic regular dynamics which could be considered as an indicator of the degree of the pureness of the field-resonator states. The regularity of the generated optical tomography disappears as the qubit- resonator detuning increases, i.e., the probability generation another pure coherent resonator states, due to the qubit-resonator interactions is very low. The intrinsic decoherence increases the mixedness of the generated filed-resonator states, leading the interference optical tomography regions to vanish.

**Figure 4:**
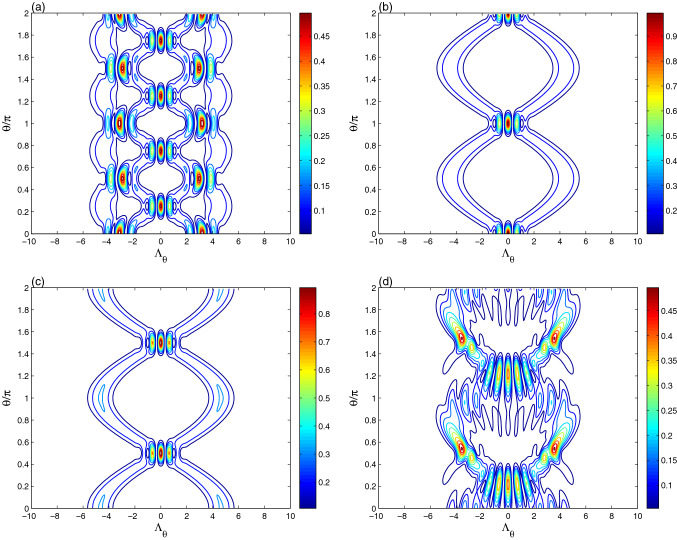
The dynamics of the optical tomography contour of the initial even coherent state for $|\alpha |^{2}=9$ with different times is plotted for $\lambda t= \frac{1}{4}\pi$ in (**a**), $\lambda t= \frac{1}{2}\pi$ in (**b**) and $\lambda t=\pi$ in (**c**). In (**d**), the optical tomography of (**c**) is displayed under the detuning effect $\delta =10\lambda$.

In the Fig. 4, for the qubit-resonator interaction, the dynamics of the optical tomography contour of the initial even coherent state at different times $\lambda t= \frac{1}{4}\pi$, $\lambda t= \frac{1}{2}\pi$ and $\lambda t=\pi$ is plotted. For the resonant case $\delta =0$ and $\lambda t= \frac{1}{4}\pi$, the contour plot illustrates that the optical tomography has more picks (which appear at high intensity) and more intersected symmetric as well as regular path. The amplitudes and the contour intensity of the generated optical ECS-tomography are smaller than those of the initial even coherent state, see Fig. 1b. At $\lambda t= \frac{1}{2}\pi$ (see Fig. 4b), the resonator reduced matrix presents a new shape for the the optical ECS-tomography with two regular sine-curve paths. It is similar to the initial even coherent state of Fig. 1b as it is expected from the dynamics of the resonator entropy, see the sub-figure of Fig. 1d. To prove the relation between the optical ECS-tomography and the resonator field entropy, the ECS-tomography is displayed at the time $\lambda t=\pi$ (in which $S_{R}(t)=0$). It should be noted that the produced ECS-tomography can be roughly restored to the initial distribution.

**Figure 5:**
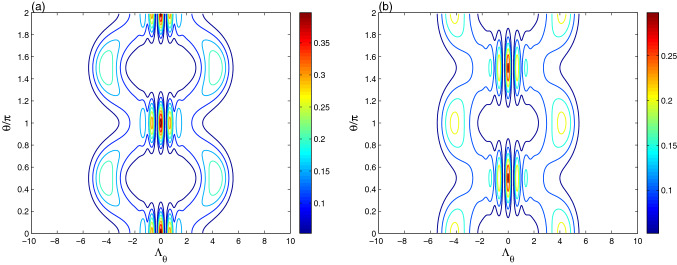
The effect of the intrinsic decoherence on the optical tomography dynamics of the Fig. 2b,c for $\gamma =0.05\lambda$.

Figure 4d confirms that the qubit-resonator detuning modifies the amplitudes, frequency, and shapes of the optical ECS-tomography.

Figure 5 shows the intrinsic decoherence effect on the tomography dynamics of the coherent states at different times $\lambda t= \frac{1}{2}\pi$ and $\lambda t=\pi$ (Fig. 4b,c). The amplitudes and the intensity of the ECS-tomography oscillations are reduced as well as the shape of the distributions is modified due to the intrinsic decoherence effect.

From Figs. 1 and 2, we note that the amplitudes, frequency, and shapes of the optical tomography of $|\alpha \rangle$ are time-dependent. Therefore, qubit-resonator interaction allows to prepare particularly different interesting states. The optical tomography can not be protected by using the coherent state as an initial state. The generated ECS-tomography may be restored to the initial distribution of the even/odd coherent state, as shown in Figs. 1 and 4. As a result, we deduce that the optical tomography protected information is reliant on the initial coherent/even-coherent resonator state.

## Conclusions

We have explored the dynamics of a quantum system formed by a qubit and a resonator coupled by a two-photon interaction. We have analyzed the optical tomography and quantum coherence dynamics of the resonator state when it is initially in a superposition of coherent states in the presence of intrinsic decoherence. The results demonstrate that owing to qubit-resonator interactions, there is a connection between the optical tomography and the generated resonator quantum coherence. We have investigated the effects of qubit-resonator detuning and intrinsic decoherence on the dynamics of optical tomography distributions for initial coherent and even coherent resonator states. When the qubit-resonator detuning and intrinsic decoherence are augmented, the amplitude and intensity, as well as the structure of the optical tomography, change substantially.

## Data Availability

The datasets used and/or analyzed during the current study available from the corresponding author (A.-B.A.M.) on request.
